# Activation of BK Channels Prevents Hepatic Stellate Cell Activation and Liver Fibrosis Through the Suppression of TGFβ1/SMAD3 and JAK/STAT3 Profibrotic Signaling Pathways

**DOI:** 10.3389/fphar.2020.00165

**Published:** 2020-03-06

**Authors:** Linli Yang, Bo Han, Man Zhang, Ya-Hui Wang, Kun Tao, Michael X. Zhu, Kunyan He, Zhi-Gang Zhang, Shangwei Hou

**Affiliations:** ^1^Key Laboratory of Systems Biomedicine, Ministry of Education, Shanghai Center for Systems Biomedicine, Shanghai Jiao Tong University, Shanghai, China; ^2^Department of General Surgery, Key Laboratory for Translational Research and Innovative Therapeutics of Gastrointestinal Oncology, Hongqiao International Institute of Medicine, Tongren Hospital, Shanghai Jiao Tong University School of Medicine, Shanghai, China; ^3^State Key Laboratory of Oncogenes and Related Genes, Shanghai Cancer Institute, Ren Ji Hospital, Shanghai Jiao Tong University School of Medicine, Shanghai, China; ^4^Department of Pathology, Tongren Hospital, Shanghai Jiao Tong University School of Medicine, Shanghai, China; ^5^Department of Integrative Biology and Pharmacology, McGovern Medical School, The University of Texas Health Science Center at Houston, Houston, TX, United States; ^6^Department of Anesthesiology, Key Laboratory for Translational Research and Innovative Therapeutics of Gastrointestinal Oncology, Hongqiao International Institute of Medicine, Tongren Hospital, Shanghai Jiao Tong University School of Medicine, Shanghai, China

**Keywords:** liver fibrosis, hepatic stellate cells, BK, KCNMA1, TGFβ1

## Abstract

Large-conductance and Ca^2+^-activated K^+^ (BK) channels are expressed in human hepatic stellate cells (HSCs), where they have roles in normal hepatic microcirculation, as well as in portal hypertension in liver cirrhosis through the regulation of contractility in activated HSCs. Nevertheless, whether BK channel activity exerts protective effects against aberrant HSC activation and hepatic fibrosis is unknown. Here, we report that BK channels are expressed in activated primary rat HSCs as well as in a human HSC line. Moreover, whole-cell K^+^ currents recorded from activated HSCs were markedly increased by exposure to rottlerin, a BK channel-specific activator, but were inhibited by treatment with the BK channel-specific inhibitor, paxilline, suggesting that BK channels are functional in activated HSCs. Overexpression but not downregulation of the BK channel pore-forming alpha subunit, KCNMA1, led to reduced migration and collagen expression in activated HSCs. Consistently, rottlerin treatment suppressed the fibrogenic cell function both *in vitro* and in CCl_4_-induced liver fibrosis *in vivo*. Microarray and pathway analysis, combined with a luciferase reporter assay and western blotting, further showed that rottlerin treatment led to a significant downregulation of the profibrotic TGFβ1/SMAD3 and JAK/STAT3 signaling pathways, both *in vitro* and *in vivo*. Our findings not only link BK channel function to profibrotic signaling pathways, but also provide evidence that BK channel activation represents a promising therapeutic strategy for the treatment of liver fibrosis.

## Introduction

Liver fibrosis is a frequent complication related to chronic liver diseases, and is associated with high morbidity and high mortality worldwide. Currently, no effective treatment is available for this condition (Trautwein et al., 2015). Hepatic fibrosis is characterized by disruption of the architectural organization of functional liver units due to excessive extracellular matrix (ECM) deposition (Trautwein et al., 2015; Schuppan et al., 2017, 2018). Several studies have demonstrated that hepatic stellate cells (HSCs; also referred to as Ito cells, fat-storing cells, lipocytes, perisinusoidal cells, and parasinusoidal cells) are important contributors to the development and progression of liver fibrosis, indicating that targeting HSC activation may be a promising therapeutic strategy for the treatment of liver fibrosis (Mederacke et al., 2013; Iwaisako et al., 2014; Trautwein et al., 2015; Higashi et al., 2017).

Under normal physiological conditions, HSCs reside in Disse’s space, where they exhibit a quiescent phenotype. However, HSCs become activated in response to a variety of profibrotic factors, such as transforming growth factor beta 1 (TGFβ1), that are generated during chronic liver injury, and exhibit phenotypes characterized by enhanced aortic smooth muscle actin (ACTA2) expression (Hernandez-Gea and Friedman, 2011; Tsuchida and Friedman, 2017; Shang et al., 2018), collagen generation, contraction, migration, and proliferation (Reynaert et al., 2002; Friedman, 2010; Page et al., 2014; Seki and Schwabe, 2015).

Ionic homeostasis is crucial for cell survival, metabolism, and immunity. In humans, large-conductance Ca^2+^- and voltage-activated K^+^ (BK) channels are expressed in a wide variety of tissues and are important for numerous physiological processes, including neuronal excitability, synaptic transmission, and regulation of vascular tone (Hoshi et al., 2013; Latorre et al., 2017). Studies have shown that BK channels are expressed in fibrotic HSCs and are involved in HSC contractility (Gasull et al., 2001), which may be important for attenuating portal hypertension in cirrhosis (Rodriguez-Vilarrupla et al., 2008). However, whether BK channels have additional roles in activated HSCs, such as proliferation, migration, and collagen synthesis, the main phenotypes associated with liver fibrosis, has not been addressed. In this study, we examined the expression pattern of BK channels in activated HSCs, and also evaluated their potential roles in HSC activation, as well as the underlying molecular mechanisms. Moreover, we assessed the antifibrotic effect of BK channel activation using both *in vitro* and *in vivo* models of fibrosis.

## Materials and Methods

### Reagents

Human recombinant TGFβ1 was purchased from Peprotech (Rocky Hill, CT, United States). Dulbecco’s modified Eagle’s Medium (DMEM), penicillin, streptomycin, and L-glutamine were obtained from Corning (NYC, NY, United States). Fetal bovine serum (FBS) was purchased from Invitrogen (NYC, NY, United States), and rottlerin and paxilline were from Sigma–Aldrich (Saint Louis, MO, United States). Antibodies against SMAD3, p-SMAD3, JAK2, p-JAK2, and β-actin were obtained from Cell Signaling Technology (Danvers, MA, United States), the anti-α-SMA (ACTA2) antibody was from Novus Biology (Centennial, CO, United States), and the anti-BK-Slo1 was purchased from Alomone Labs (Jerusalem, Israel). Alexa Fluor 680-conjugated anti-mouse and anti-rabbit secondary antibodies were purchased from Life Technologies (Eugene, OR, United States). Alanine transaminase (ALT) and aspartate transaminase (AST) detection reagents were obtained from Shanghai Shensuo UNF Medical Diagnostic Articles Corporation (Shanghai, China).

### HSC Isolation and Cell Culture

Isolation and culture of liver HSCs from adult male Sprague–Dawley rats (body weight, 500–600 g) were performed as previously described (Seki et al., 2007). Briefly, rats were anesthetized with 1 mL of ketamine (10%) and 50 μL of xylazin (2%) per kg body weight according to an institutionally approved protocol (Shanghai Jiao Tong University Institutional Animal Care and Use Committee). The peritoneum was then exposed and the liver quickly perfused via the portal vein with 40 mL of Hank’s balanced salt solution (HBSS) without Ca^2+^ and Mg^2+^ at a flow rate of 10 mL/min. Next, the liver was perfused with 30 mL (1 mg/mL) of pronase E followed by 30 mL (0.25 mg/mL) of collagenase H. The liver was carefully separated and transferred to HBSS (with Ca^2+^ and Mg^2+^) containing pronase E (0.2 mg/mL) and collagenase H (0.25 mg/mL), and the liver lobes were cut into small pieces and digested for 20 min at 37°C with continuous stirring. The hepatocytes were centrifuged at 50 × *g* and the cell suspension was filtered through 100-μm cell strainers, washed four times with HBSS (with Ca^2+^ and Mg^2+^), and subjected to Nycodenz (13.2%) gradient centrifugation at 1400 × *g* for 20 min at 4°C. HSCs were concentrated in the interphase as a white, ring-like structure. After harvesting and washing with HBSS, the HSCs were seeded on uncoated plastic tissue culture dishes in DMEM supplemented with 10% FBS, 4 mM L-glutamine, 100 U/mL penicillin, and 100 μg/mL streptomycin. The first change of medium was performed 24 h after seeding. Quiescent HSCs were used for experiments 2–3 days after seeding, and activated HSCs were used 9–10 days after seeding. The human hepatic stellate cell line LX2 was cultured under the same conditions as for the rat HSCs. TGFβ1 (4 ng/mL) was added to the culture medium to test protein phosphorylation of HSCs at different time points or to differentiate LX2 cells into activated myofibroblast-like cells. All the cells were grown and maintained at 37°C and 5% CO_2_ in a humidified incubator.

### RNA Isolation and Quantitative PCR (qPCR)

Total RNA was extracted from primary rat HSCs and LX2 cells using Trizol reagent (Invitrogen, Carlsbad, CA, United States). cDNA was synthesized using a reverse transcription kit (TaKaRa, Dalian, China). Real-time PCR was performed using 2 × SYBR Green qPCR Master Mix (Bimake, Houston, TX, United States). RNA was amplified using the primers indicated in Table 1. Relative mRNA expression levels were calculated and normalized to the levels of endogenous beta-actin in the same samples. Real-time PCR was performed in triplicate for each experiment and repeated at least three times and data was analyzed by 2^–△△*CT*^ method.

**TABLE 1:** List of primers for qPCR.

**Gene**	**Forward primer (5′–3′)**	**Reverse primer (5′–3′)**
**Human**		
*ACTB*	GTACGCCAACACAGTGCTG	CGTCATACTCCTGCTTGCTG
*ACTA2*	CGTGCTGGACTCTGGAGATG	GCCCATCAGGCAACTCGTAA
*KCNMA1*	TCTTTGCTCTCAGCATCGGTG	CCGCAAGCCGAAGTAGAGAAG
*COL1A1*	CGGTGTGACTCGTGCAGC	ACAGCCGCTTCACCTACAGC
*COL1A2*	TCAAACTGGCTGCCAGCAT	CAAGAAACACGTCTGGCTAGG
*CCL2*	TCGCGAGCTATAGAAGAATCA	TGTTCAAGTCTTCGGAGTTTG
**Rat**		
*Actb*	GTACGCCAACACAGTGCTG	CGTCATACTCCTGCTTGCTG
*Acta2*	TGTGCTGGACTCTGGAGATG	GATCACCTGCCCATCAGG
*Kcnma1*	AAGGAATGCATCTTGGCGTC	TCTGTGTCAGGGTCATCATCATC

### Cell Viability Assay

Cells were seeded in 96-well plates at a density of 5 × 10^3^ cells per well overnight, and then exposed to chemicals at the indicated concentrations for 48 h. Subsequently, 10 μL of MTS was added to each well and incubated for a minimum of 1 h. Measurements were made in accordance with the manufacturer’s instructions (Promega Corp., Madison, WI, United States). Metabolic activity was recorded as the relative colorimetric change measured at 492 nm. Each experiment was performed at least three times.

### EdU Cell Proliferation Assay

The EdU cell proliferation assay was performed according to the manufacturer’s instructions. Briefly, LX2 cells were transfected with *KCNMA1*-expressing plasmids or KCNMA1 siRNA for 48 h, and then plated in 96-well plates and incubated for 24 h. After incubation, the EdU solution (10 mM) was added directly to the culture medium and incubated for a further 30 min to ensure capture of most of the proliferating cells. The cells were subsequently fixed in 4% paraformaldehyde. Incorporation of EdU was performed by incubating the fixed cells with 2% bovine serum albumin (BSA) in PBS for 30 min and Alexa Fluor 488 for a further 30 min under Cu(I)-catalyzed click reaction conditions. The cells were washed with PBS and counterstained with Hoechst 33342 (1:1000) in PBS before detection by fluorescence microscopy using ImageXpress^®^ Micro 4 (Molecular Devices, San Jose, CA, United States).

### Western Blot Assay

Cells were lysed on ice with lysis buffer (50 mM Tris–HCl, 150 mM NaCl, 1% NP-40, 0.5% sodium deoxycholate, and 0.1% SDS, pH 7.5) in the presence of a protease inhibitor mixture and phosphatase inhibitors (both from Roche, Mannheim, Germany). Protein concentrations were measured using the BCA protein assay kit (Thermo Fisher Scientific, Rockford, IL, United States). Samples were added to 5 × SDS loading buffer, boiled for 10 min, and loaded onto 10% polyacrylamide-SDS gels. The proteins were then transferred to PVDF membranes (Millipore, Billerica, MA, United States) at 300 mA for different durations, and the membranes were incubated in 5% BSA for 1 h at room temperature. After incubation with primary antibodies (Tables 2, 3) overnight at 4°C, the membranes were washed three times (10 min each) in TBS containing 0.1% Tween 20 (TBST) and then incubated with peroxidase- or fluorophore-conjugated secondary antibodies for 1 h in TBST at room temperature. The blots were detected by the ProteinSimple system (San Jose, CA, United States) with an ECL substrate or a LiCor imaging system (Lincoln, NE, United States).

**TABLE 2:** List of the KCNMA1 siRNA sequences.

**ID**	**Sense (5′–3′)**	**Antisense (5′–3′)**
siRNA1	CUGGCAGAGUCCUGGUUGUTT	ACAACCAGGACUCUGCCAGUC
siRNA2	GGGUCUGUCCUUCCCUACUTT	AGUAGGGAAGGACAGACCCAC

**TABLE 3:** List of antibodies for western blotting analysis.

**Antibodies**	**Source (catalog no.)**	**Dilution (×)**
β-Actin	Cell signaling technology (4967)	1000
SMAD3	Cell signaling technology (9523)	1000
p-SMAD3	Cell signaling technology (9520)	1000
KCNMA1	Allomone labs (APC-107)	500
ACTA2	Novus (NBP1-97722)	2000
p-TGFBR1	Thermal scientific (PA5-40298)	1000
p-TGFBR2	Thermal scientific (PA5-37755)	1000
**List of antibodies for immunofluorescence and immunohistochemistry**		
KCNMA1	Allomone labs (APC-107)	100
ACTA2	Novus (NBP2-33006)	500

### Electrophysiology and Analysis

Macroscopic currents were recorded from HSCs in a whole-cell configuration using an EPC-10 amplifier controlled by PatchMaster software (HEKA Electronics, Ludwigshafen/Rhein, Rheinland-Pfalz, Germany) as previously described (Han et al., 2016). Patch pipettes were prepared from borosilicate glass (Warner, Hamden, CT, United States) and had a resistance of 2–5 M Ω when filled with a pipette solution containing 140 mM K-aspartate, 4.3 mM CaCl_2_, 2.06 mM MgCl_2_, 5 mM EGTA, and 10 mM HEPES, pH 7.2. Free Ca^2+^ concentrations were calculated to be 1 μM using the Maxchelator program (Stanford University). The standard external recording solution comprised 150 mM sodium aspartate, 5 mM KCl, 2 mM CaCl_2_, 1 mM MgCl_2_, 10 mM glucose, and 10 mM HEPES, pH 7.4. Currents were elicited every 5 s by 200 ms voltage ramps from −100 to 80 mV. Data analysis and curve fitting were performed using FitMaster (HEKA Electronics, Ludwigshafen/Rhein, Rheinland-Pfalz, Germany) and figures were prepared with Origin 8.5 (OriginLab, Northampton, MA, United States).

### Plasmid and siRNA Transfection

Overexpression or downregulation of human KCNMA1 (AAB65837) was accomplished by transient transfection of plasmids containing the KCNMA1 gene or siRNA targeting KCNMA1 into LX2 cells for 48–72 h. Lipofectamine^TM^ 2000 (Thermo Fisher Scientific, Carlsbad, CA, United States) was used for transient transfection according to the manufacturer’s instructions.

### Cell Migration Assay

The migration capacity of human LX2 cells and activated primary rat HSCs was measured by transwell assay (Corning, NY, United States) according to the manufacturer’s instructions. Briefly, 5 × 10^4^ cells were seeded into the upper chamber of transwell inserts. DMEM supplemented with 10% (v/v) FBS was added to the bottom chamber. Cells were incubated at 37°C and allowed to migrate for 16 h for LX2 cells and 12 h for activated rat HSCs, respectively. At the designated time points, cells were fixed in 4% paraformaldehyde and stained with 0.2% crystal violet, and non-migrating cells on the upper chamber surface were removed with cotton swabs. The number of cells on the lower surface was counted in five random fields under a light microscope.

### Immunofluorescence and Immunohistochemistry

For immunostaining, cells were fixed in 4% (w/v) paraformaldehyde in PBS for 1 h at room temperature. After fixation, the cells were rinsed with PBS and then permeabilized with 0.3% Triton X-100. Non-specific binding was blocked with 10% (w/v) BSA for 1 h at room temperature. The cells were then incubated with the respective primary antibodies (Table 3) overnight at 4°C. After extensive washing, cells were incubated with secondary antibodies for 1 h at room temperature, and nuclei were stained with DAPI. Confocal microscopy and image acquisition were carried out using a Nikon A1Si laser scanning confocal microscope. For liver section staining, paraffin sections (5 μm) were deparaffinized, rehydrated through a graded ethanol series, incubated with 0.3% hydrogen peroxide for 30 min, and blocked with goat serum. The sections were then incubated with the anti-ACTA2 antibody overnight at 4°C, followed by labeling with a horseradish peroxidase (HRP)-conjugated antibody (Thermo Fisher Scientific, Eugene, OR, United States) at room temperature for 1 h. Staining was visualized with DAB (Gene Tech, Shanghai, China). All sections were observed and imaged under a microscope (Carl Zeiss, Jena, Germany).

### Rat Model of Liver Fibrosis

Eighteen pathogen-free male Sprague–Dawley rats (∼200 g) were randomly assigned to three groups, namely, control, fibrosis (CCl_4_-treated), and rottlerin-treated groups. The animals in the fibrosis group were injected intraperitoneally with 0.2 mL/100 g of a CCl_4_/olive oil mix (1:1) twice weekly, at equal intervals, for 8 weeks, as previously described (Constandinou et al., 2005). The rats in the rottlerin-treated group were injected intraperitoneally with rottlerin (2.5 mg/kg body weight) 2 weeks after the first CCl_4_ injection. All the animal experiments were conducted according to protocols approved by the Shanghai Jiao Tong University Institutional Animal Care and Use Committee.

### Luciferase Reporter Assay

LX2 cells at 60–70% confluence were plated in 96-well plates and transiently transfected with 0.15 μg of the pGL4.48[luc2P/SBE/Hygro] vector (Promega, Madison, WI, United States) and 0.015 μg of the *Renilla* luciferase control reporter vector (Promega) using FuGENE 6 transfection reagent (Promega) according to the manufacturer’s instructions. SBE means SMAD binding element. Co-transfection with a vector expressing *Renilla* luciferase under the control of the SV40 early enhancer/promoter was used to normalize transfection efficiency. After 24 h of transfection, LX2 cells were treated with TGFβ1 (4 ng/mL) for 24 h. In the rottlerin-treated group, LX2 cells were treated with TGFβ1 (4 ng/mL) for 12 h and then co-treated with rottlerin (3 μM) for 12 h. The cells were collected and subjected to a dual-luciferase reporter assay (E1910, Promega). For each sample, firefly luciferase activity was normalized to that of *Renilla* luciferase.

### Microarray Analysis

Comparative microarray analysis was performed using the Affymetrix GeneChip^®^ Rat Genome 230 2.0 platform. Each treatment had three replicates, resulting in three independent microarrays for each treatment and controls (9 arrays in total). Genes showing differences in probe intensity of 1.5-fold or more were further analyzed. Differentially expressed genes were classified into their Gene Ontology (GO)^1^ functional categories using R/Bioconductor (Bioconductor).^2^ Cellular pathway association was analyzed according to the Kyoto Encyclopedia of Genes and Genomes (KEGG) database^3^ and pathway maps were analyzed according to BioCarta.^4^ Microarray data are available via the GEO accession number GSE139994.

### Statistical Analysis

Data are expressed as means ± SEM. Significance was evaluated using either paired or unpaired Student *t*-test or one-way AONVA analysis if the data showed normal distribution and *homogeneity of variance* which were evaluated by Shapiro-Wilk test and Levene’s test, respectively. Otherwise, Wilcoxon signed-rank test was used to analyze the paired data and *Mann-Whitney U test and Kruskal-Wallis H test were used to analyze unpaired data for two and multiple groups*, respectively. A *p*-value < 0.05 was considered significant.

## Results

### BK Channels Are Expressed and Functional in Activated HSCs

**FIGURE 1:**
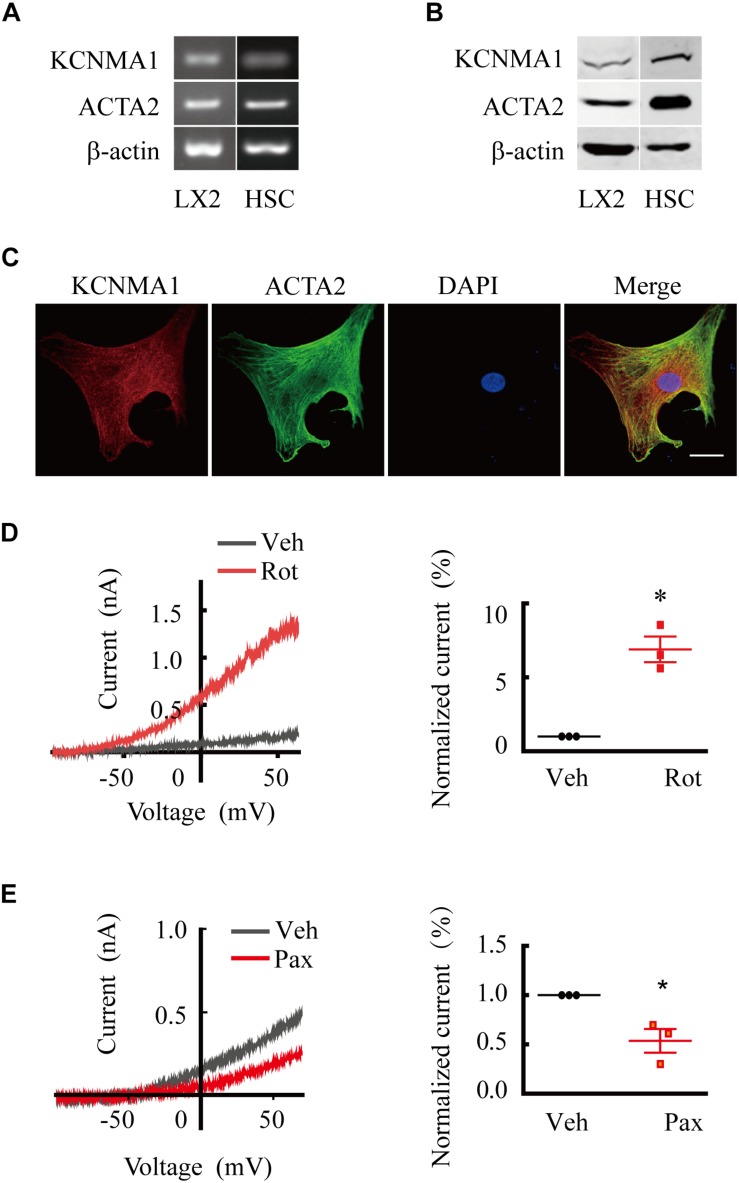
Large-conductance and Ca^2+^-activated K^+^ (BK) channels are expressed and functional in activated hepatic stellate cells (HSCs). **(A,B)** Representative RT-qPCR **(A)** and western blots **(B)** showing the expression of ACTA2 and the BK channels alpha subunit KCNMA1 in activated human LX2 cells treated with transforming growth factor beta 1 (TGFβ1) and spontaneously activated primary rat HSCs *in vitro*. **(C)** Representative immunofluorescence images of KCNMA1 and ACTA2 in activated primary rat HSCs (Scale bars, 25 μm). **(D,E)** Representative whole-cell K^+^ current traces and the normalized current recorded from activated HSCs before and after treatment with rottlerin (Rot) and paxilline (Pax), as indicated at 1 μM internal Ca^2+^. The whole-cell currents were elicited by 1 s voltage ramps from −100 to 80 mV. Rottlerin (1 μM) and paxilline (10 μM) were freshly prepared from stock solutions and the final DMSO concentration was 0.1% (*p < 0.05 compared with the vehicle group, *n* = 3).

To determine whether BK channels were expressed in activated HSCs, we examined the mRNA and protein expression of the BK channel pore-forming alpha subunit KCNMA1 in the LX2 cell line (Xu et al., 2005), a partially activated human HSC line that can be fully activated by TGFβ1 (Tsuchida and Friedman, 2017). We found that KCNMA1 was expressed at both the mRNA and protein level in TGFβ1-activated LX2 cells (Figures 1A,B) that showed robust expression of ACTA2, a marker for activated HSCs (Tsuchida and Friedman, 2017; Shang et al., 2018). To investigate whether KCNMA1 is also expressed in activated native HSCs, rat primary HSCs were isolated and spontaneously activated in serum-containing medium following 8–10 days of continuous culture on uncoated plastic. These spontaneously activated HSCs can faithfully recapitulate the major *in vivo* characteristic phenotypes of activated HSCs (Friedman et al., 1989). We found that KCNMA1 was expressed in primary activated HSCs at the level of both mRNA and protein (Figures 1A,B), while immunostaining indicated that KCNMA1 was distributed throughout the cell membrane of activated HSCs (Figure 1C).

To investigate whether BK channels in activated HSCs were functional, we performed whole-cell patch-clamp recordings combined with pharmacological evaluation. We found that whole-cell K^+^ currents from activated HSCs showed apparent voltage-dependent outward rectification properties. Extracellular application of rottlerin, a BK channel-specific activator, markedly increased the outward K^+^ current (Figures 1D,E), whereas treatment with paxilline, a BK channel-specific blocker, elicited the opposite effect, demonstrating that BK channels were functional in activated HSCs.

### Overexpression of the BK Channel Alpha Subunit Inhibits Migration and the Expression of Collagen Genes in HSCs

**FIGURE 2:**
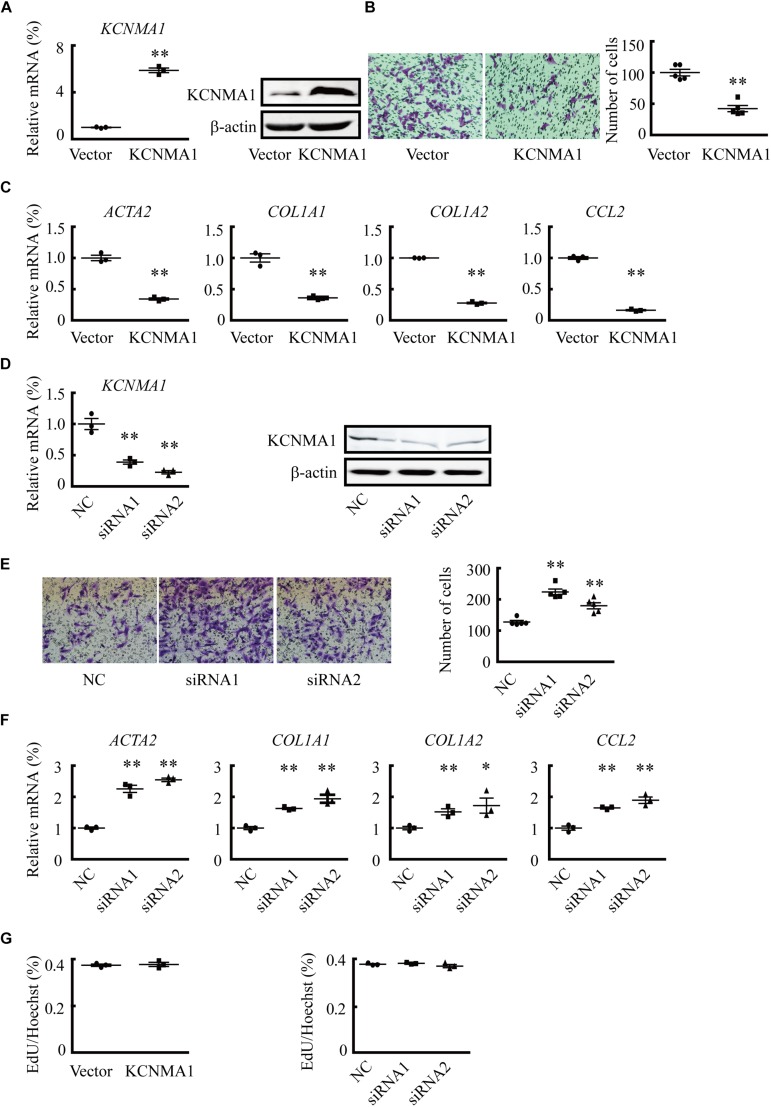
Overexpression of the BK channel alpha subunit (KCNMA1) inhibits hepatic stellate cell (HSC) migration and fibrosis-related gene expression. **(A)** Validation of KCNMA1 overexpression by qPCR (*left*) and western blotting (*right*). **(B)** Representative images of cells (*left*) and average number of migrated cells (*right*) showing the inhibitory effects of transient KCNMA1 overexpression on LX2 cell migration. **(C)** Normalized mRNA expression of aortic smooth muscle actin (*ACTA2*), collagen type I alpha 1 chain (*COL1A1*), *COL1A2*, and C-C motif chemokine ligand 2 (*CCL2*) in LX2 cells pretreated with TGFβ1 after transfection with *KCNMA1*-expressing plasmids. **(D)** Detection of KCNMA1 by qPCR (*left*) and western blotting (*right*) after siRNA transfection. **(E)** Representative images (*left*) and averaged number of migrated cells (*right*) showing the inhibitory effects of knocking down KCNMA1 on LX2 cell migration. **(F)** Normalized mRNA expression levels of *ACTA2*, *COL1A1*, *COL1A2*, and *CCL2* in LX2 cells pretreated with TGFβ1 after transfection with siRNA targeting KCNMA1. **(G)** Ratio of EdU-positive cells to Hoechst 33342-stained nuclei after transfection of *KCNMA1*-expressing plasmids (*left*) or KCNMA1 siRNA (*right*) (^∗^*p* < 0.05, ^∗∗^*p* < 0.01 compared with the vector or negative control (NC) group.

BK channels are associated with cell migration in multiple cell types (Toro et al., 2014). To explore the potential roles of BK channels in migration, a prominent feature of activated HSCs (Tsuchida and Friedman, 2017), we overexpressed the BK channel pore-forming alpha subunit, KCNMA1, by transfecting *KCNMA1*-expressing vectors into LX2 cells. The results showed that overexpression of KCNMA1 (Figure 2A) decreased LX2 cell migration (Figure 2B). Furthermore, *ACTA2*, collagen type I alpha 1 chain (*COL1A1*), *COL1A2*, and C-C motif chemokine ligand 2 (*CCL2*) mRNA expression levels were also decreased (Figure 2C). In contrast, downregulating KCNMA1 expression (Figure 2D) through siRNA transfection increased the migration rates of LX2 cells (Figure 2E), as well as the expression of *ACTA2*, *COL1A1*, *COL1A2*, and *CCL2* (Figure 2F), indicating that enhancement of BK channel function could reverse HSC activation. However, neither overexpression nor knockdown of KCNMA1 affected HSC proliferation, as evidenced by the results of EdU staining (Figure 2G).

### Pharmacological Activation of BK Channels Inhibits HSC Migration and Collagen Synthesis

**FIGURE 3:**
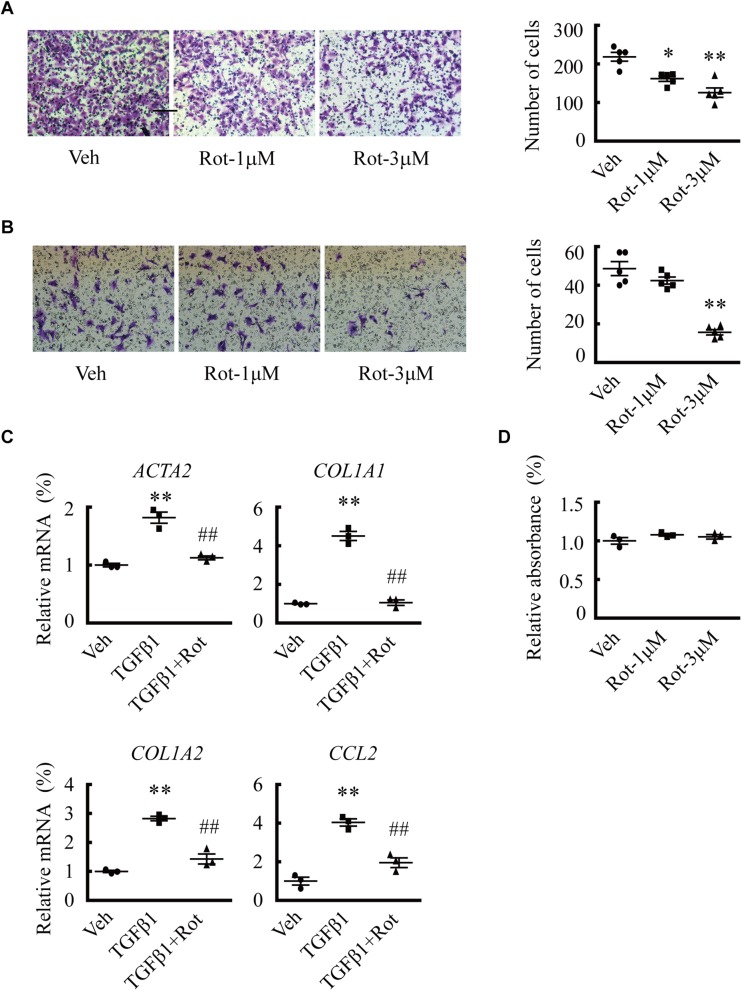
Upregulation of large-conductance and Ca^2+^-activated K^+^ (BK) channel activity by rottlerin inhibits hepatic stellate cell (HSC) migration and fibrosis-related gene expression *in vitro*. **(A,B)** Representative images (*left*) and averaged number of migrated cells (*right*) showing the inhibitory effects of rottlerin (Rot), a BK channel activator, on the migration of human LX2 cells **(A)** and activated primary rat HSCs **(B)** at the indicated concentrations (^∗^*p* < 0.05, ^∗∗^*p* < 0.01 compared with vehicle). **(C)** Relative mRNA expression levels of *ACTA2*, *COL1A1*, *COL1A2*, and *CCL2* in human LX2 cells with the indicated treatments for 24 h. **(D)** Normalized cell viability of LX2 cells measured under the indicated conditions (^∗∗^*p* < 0.01 compared with vehicle, ^##^*p* < 0.01 compared with the TGFβ1-treated group).

We then examined the effects of rottlerin treatment on phenotypic changes of activated HSCs. The results showed that rottlerin decreased the migration of both human LX2 cells (Figure 3A) and spontaneously activated rat HSCs (Figure 3B) at micromolar concentrations. In addition, rottlerin treatment also suppressed TGFβ1-induced expression of *ACTA2*, *COL1A1*, *COL1A2*, and *CCL2* (Figure 3C), the expression of which is greatly increased in HSCs of liver fibrosis patients. Accordingly, rottlerin-induced BK channel activation did not affect the proliferation of LX2 cells (Figure 3D).

### BK Channel Activation Ameliorates CCl4-Induced Fibrosis in Rats

**FIGURE 4:**
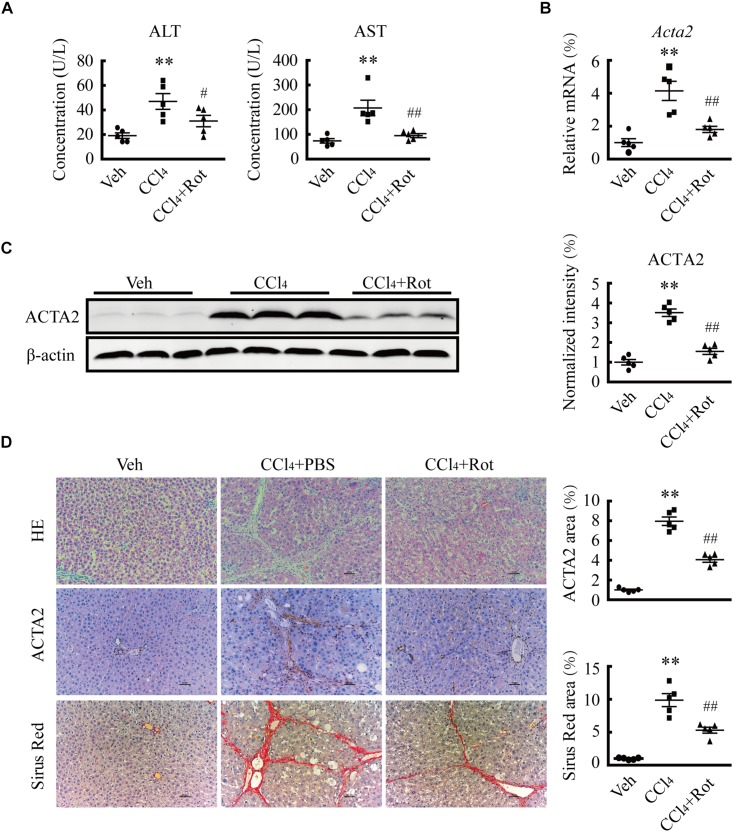
Treatment with the large-conductance and Ca^2+^-activated K^+^ (BK) channel activator rottlerin ameliorates CCl_4_-induced liver fibrosis *in vivo.* **(A)** Serum levels of alanine transaminase (ALT) and aspartate transaminase (AST) from rats in the indicated groups (^∗∗^*p* < 0.01 compared with the vehicle (Veh) group, ^#^*p* < 0.05 and ^##^*p* < 0.01 compared with the CCl_4_-treated group, *n* = 5). **(B)** Relative mRNA levels of *ACTA2* in the liver tissue of rats subjected to the indicated treatments (^∗∗^*p* < 0.01 compared with the Veh group, ^##^*p* < 0.01 compared with the CCl_4_-treated group, *n* = 5). **(C)** Representative western blots (*left*) and normalized intensity (*right*) of the indicated ACTA2 protein levels in rats subjected to the indicated treatments (^∗∗^*p* < 0.01 compared with the Veh group, ^##^*p* < 0.01 compared with the CCl_4_-treated group, *n* = 5). **(D)** Representative images of liver sections stained with hematoxylin and eosin (H&E), anti-ACTA2 antibody, and Sirius Red (*left*) and quantification of the positive area (*right*), in rats subjected to the indicated treatments (^∗∗^*p* < 0.01 compared with the Veh group, ^##^*p* < 0.01 compared with the CCl4-treated group, scale bar: 50 μm, *n* = 5).

The results of the *in vitro* experiments suggested that augmentation of BK channel activity likely facilitates the reversion of activated HSCs to a quiescent state, which may have a protective effect against liver fibrosis. To assess whether increasing BK channel function elicits antifibrotic effects *in vivo*, we tested the effect of rottlerin administration on CCl_4_-induced liver fibrosis in rats, in which myofibroblasts are mainly derived from activated HSCs (Iwaisako et al., 2014). As expected, the levels of ALT and AST, two typical serum markers of liver injury (Figure 4A), the expression of ACTA2, and levels of collagen deposition all were increased in rats with CCl_4_-induced liver fibrosis (Figures 4B–D). Interestingly, rottlerin treatment markedly suppressed these CCl_4_-induced effects (Figures 4A–D), indicating that rottlerin can elicit a significant improvement in the pathology of fibrosis, as well as in liver function.

### BK Channel Activation Attenuates Liver Fibrosis Through Inhibition of the TGFβ1/SMAD3 and JAK2/STAT3 Signaling Pathways

**FIGURE 5:**
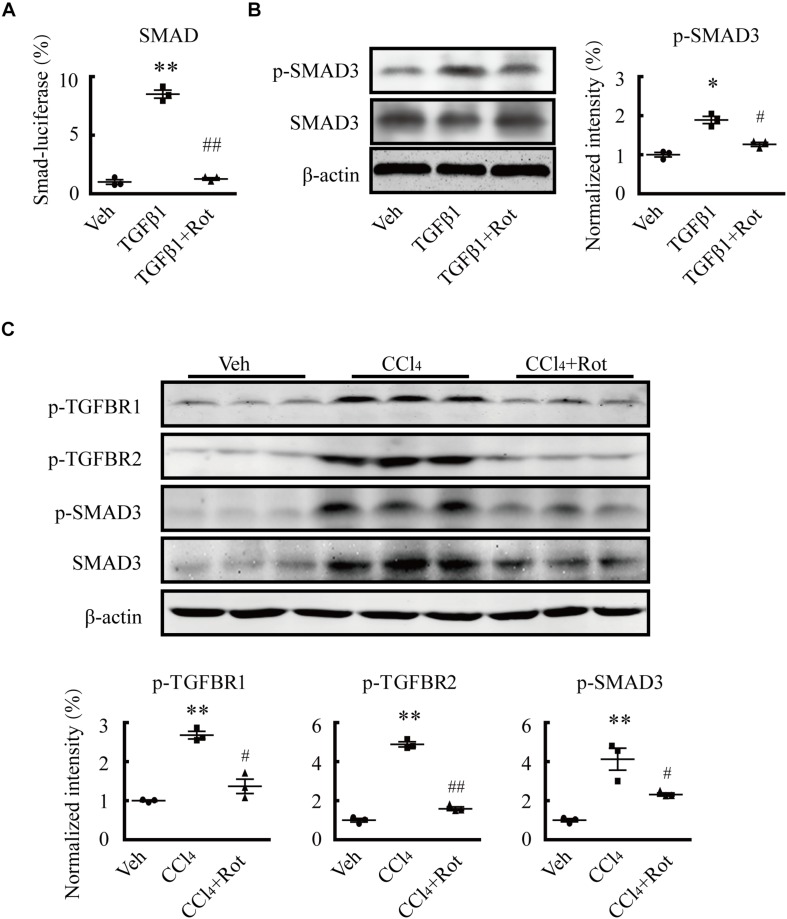
Rottlerin exerts antifibrotic effects through inhibition of the TGFβ1/SMAD3 signaling pathway. **(A)** SMAD activity as measured by luciferase reporter assay using reporter vectors PGL4.48[luc2P/SBE/Hygro] in LX2 cells with the indicated treatments. SBE means SMAD binding element (**p < 0.01 compared with the vehicle (Veh) group, ^##^*p* < 0.01 compared with the TGFβ1-treated group; *n* = 3). **(B)** Representative western blots (*left*) and normalized intensity (*right*) of the p-SMAD3 levels in primary hepatic stellate cells (HSCs) treated as indicated. Phosphorylation levels of the SMAD3 protein were measured in serum-starved HSCs treated with TGFβ1 (4 ng/mL) for 1 h. Rottlerin (3 μM) was applied to the medium for 6 h before TGFβ1 application (^∗^*p* < 0.05 compared with the Veh group, ^#^*p* < 0.05 compared with the TGFβ1-treated group, *n* = 3). **(C)** Representative western blots (*left*) and normalized intensity (*right*) of the levels of total and/or phosphorylated proteins, as indicated, in liver tissues from vehicle, CCl_4_, and rottlerin-treated rats with CCl_4_-induced fibrosis (**p < 0.01 compared with the Veh group, ^#^*p* < 0.05, and ^##^*p* < 0.01 compared with the CCl_4_-treated group, *n* = 3).

**FIGURE 6:**
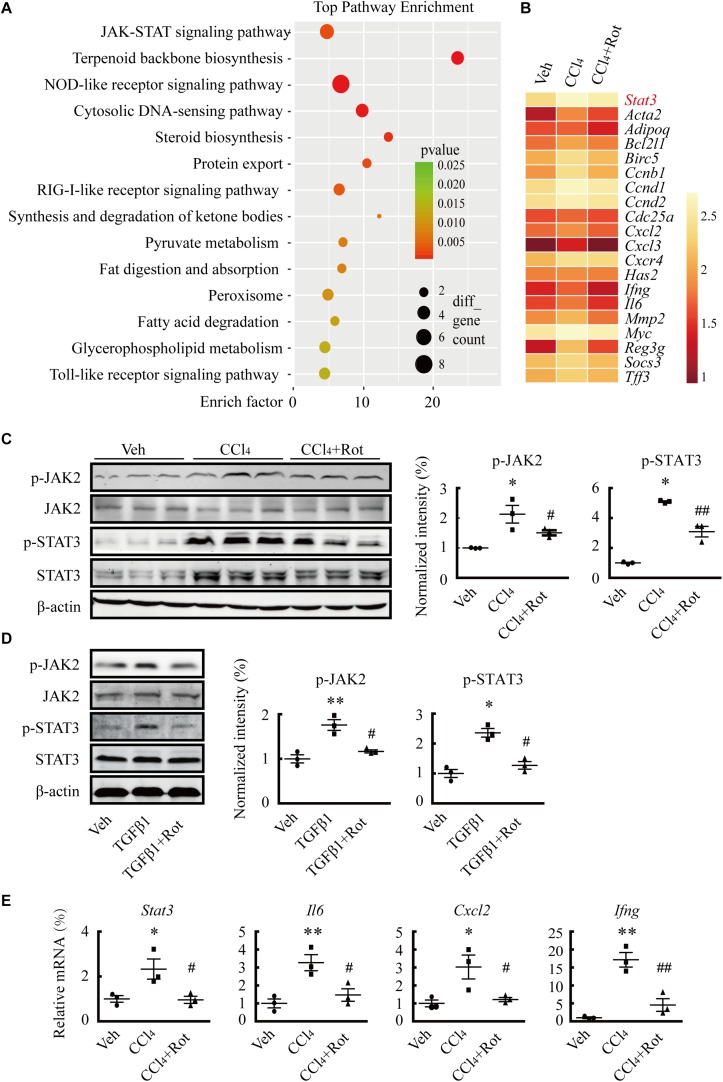
Rottlerin treatment suppresses the JAK/STAT3 signaling pathway. **(A)** KEGG (Kyoto Encyclopedia of Genes and Genomes) enrichment analysis showing the signaling pathways potentially involved in the antifibrotic effects of rottlerin in rats with CCl_4_-induced liver fibrosis (*n* = 3). **(B)** cDNA microarray showing STAT3 and its downstream target genes in liver tissues of rats treated with vehicle, CCl_4_, or rottlerin, respectively. The average signal intensity was log2-transformed. **(C)** Representative western blots (*left*) and normalized intensity (*right*) of the total and/or phosphorylated proteins as indicated in liver tissues from vehicle, CCl_4_, and rottlerin + CCl_4_-treated rats (^∗^*p* < 0.05 compared with the Veh group, ^#^*p* < 0.05 and ^##^*p* < 0.01 compared with the CCl_4_-treated group, *n* = 3). **(D)** Representative western blots (*left*) and normalized intensity (*right*) of total and phosphorylated JAK2 and STAT3 in primary hepatic stellate cells (HSCs) treated as indicated. The phosphorylation levels of JAK2 and STAT3 were measured in serum-starved HSCs treated with TGFβ1 (4 ng/mL) for 4 h for. Rottlerin (3 μM) was applied to the medium for 6 h before TGFβ1 application (^∗^*p* < 0.05 and ^∗∗^*p* < 0.05 compared with the Veh group, ^#^*p* < 0.05 compared with the TGFβ1-treated group, *n* = 3). **(E)** mRNA levels of *Stat3* and its downstream targets interleukin 6 (*Il6*), C-X-C motif chemokine ligand 2 (*Cxcl2*), and interferon gamma (*Ifng*) after rottlerin treatment in fibrotic rats (^∗^*p* < 0.05 and ^∗∗^*p* < 0.01 compared with the Veh group, ^#^*p* < 0.05 and ^##^*p* < 0.01 compared with the CCl_4_-treated group, *n* = 3).

Several studies have shown that TGFβ1 plays pivotal roles in HSC activation and hepatic fibrosis (Koyama and Brenner, 2017; Tsuchida and Friedman, 2017). To illustrate the underlying molecular mechanisms of the antifibrotic effect of BK channel activation, we first examined whether the canonical SMAD3-dependent TGFβ1 signaling pathway was involved in these processes. We found that rottlerin treatment completely blocked the TGFβ1-stimulated increase in SMAD transcriptional activity in LX2 cells, as determined by a luciferase reporter assay (Figure 5A). Western blotting results further showed that rottlerin treatment partially reduced the enhanced levels of phosphorylated, but not total, SMAD3 in TGFβ1-treated HSCs (Figure 5B), indicating that rottlerin specifically affected the phosphorylated form of SMAD3. In CCl_4_-treated rats, rottlerin administration also greatly suppressed the increased levels of total and phosphorylated SMAD3, as well as the phosphorylation of TGFβ receptor types I and II (Figure 5C), suggesting that rottlerin exerts its antifibrotic effects through the suppression of the canonical TGFβ1 signaling pathway *in vivo*. To address whether other mechanisms were also involved, we used mRNA microarray to identify differentially expressed genes in the liver tissue of rats administered vehicle, CCl_4_, or Rottlerin + CCl_4_ treatments. Pathway analysis indicated that multiple signaling pathways, including the JAK/STAT3 pathway, were involved in the rottlerin-associated antifibrotic effects *in vivo* (Figures 6A,B). As crosstalk between SMAD3 and STAT3 in numerous biological functions is well documented, and because TGFβ1 can activate STAT3, which also has a role in liver fibrosis (Tang et al., 2017; Itoh et al., 2018; Xiang et al., 2018), we hypothesized that STAT3 may contribute to the antifibrotic effects of rottlerin. Western blot results showed that rottlerin treatment could reverse the increased levels of phosphorylated JAK2, as well as the levels of total and phosphorylated STAT3, in rats with CCl_4_-induced fibrosis (Figure 6C). In addition, rottlerin treatment also attenuated the levels of phosphorylated JAK2 and STAT3 in primary activated HSCs stimulated by TGFβ1 (Figure 6D), as well as the mRNA expression of *Il6*, *Cxcl2*, and *Ifng* (Figure 6E), downstream target genes of STAT3. Combined, these results provide strong evidence that rottlerin treatment inhibits the TGFβ1/SMAD3 and JAK/STAT3 signaling pathways *in vivo*.

## Discussion

**FIGURE 7:**
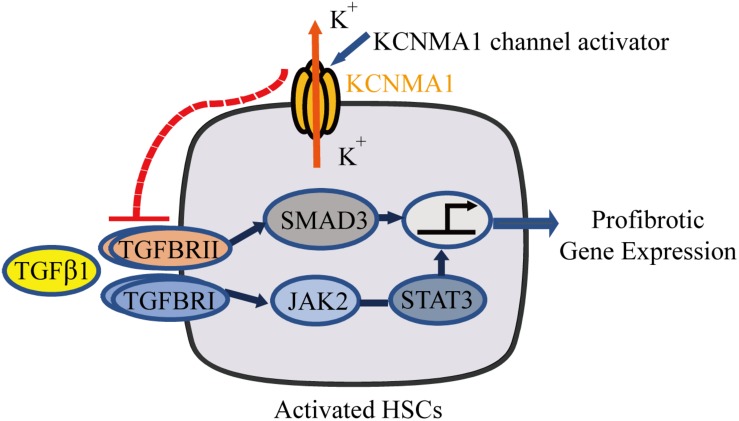
Schematic diagram showing that upregulation of large-conductance and Ca^2+^-activated K^+^ (BK) channel activity in activated hepatic stellate cells (HSCs) exerts an antifibrotic effect through suppressing of the profibrotic TGFβ1 signaling pathway.

In this study, we demonstrated that BK channels are expressed in activated HSCs and exert an antifibrotic effect through the suppression of TGFβ-associated profibrotic signaling. Consistent with these results, the BK channel activator rottlerin showed a marked antifibrotic effect, both *in vitro* and *in vivo*. Together, our results suggest that BK channel activation represents a promising therapeutic strategy for the treatment of liver fibrosis (Figure 7).

In addition to BK channels, intermediate-conductance Ca^2+^-activated K^+^ channels (KCa3.1, also known as KCNN4), have also been reported to be involved in the pathology of hepatic fibrosis. For example, the expression of KCa3.1 was reportedly upregulated in a bile duct ligation model of liver fibrosis, while exposure to the KCa3.1 channel inhibitor TRAM-34 attenuated HSC proliferation, TGFβ1-induced HSC activation, as well as portal hypertension, suggestive of a profibrotic role for these channels (Freise et al., 2015). In contrast, a different study reported that increased KCa3.1 expression was predominantly observed in hepatocytes, but not in HSCs, and appeared to exert an antifibrotic and protective role during liver injury (Sevelsted Moller et al., 2016). Meanwhile, inhibition of KCa3.1 was shown to aggravate fibrotic symptoms in a CCl_4_-induced mouse model of liver fibrosis (Sevelsted Moller et al., 2016), in which more HSC differentiation into myofibroblast was observed compared with that in a bile duct ligation model (Iwaisako et al., 2014). In our study, whole-cell current recordings from activated HSCs showed a prominent outward rectification property resembling the K^+^ current from the BK channel, but not the KCa3.1 channel. Moreover, the current characterized by rottlerin activation and paxilline blockade *in vitro*, as well as inhibition of fibrosis mediated through BK channel activation *in vivo*, further support that BK channels may play more important roles than KCa3.1 channels in hepatic fibrosis, at least in models of CCl_4_-induced liver fibrosis.

Several studies have shown that BK channels are expressed in human HSCs and potentially attenuate the portal hypertension that typically occurs in the late stage of liver fibrosis (Gasull et al., 2001; Rodriguez-Vilarrupla et al., 2008). We demonstrated that BK channel activation by a channel-specific activator suppresses HSC activation and fibrosis, indicating that BK channels are important in the early stage of this condition. Our study supports that the BK channel exerts its antifibrotic effects through the inhibition of the canonical TGFβ1/SMAD3 signaling pathway, as evidenced by the notable reduction in SMAD3 and STAT3 phosphorylation levels induced by rottlerin treatment, both *in vitro* and *in vivo*. Activation of BK channels either through overexpression of the BK channel pore-forming alpha subunit, KCNMA1, or by pharmacological means, greatly inhibited the migration capacity of activated HSCs. This could be explained, at least in part, by the rottlerin treatment-induced decrease in the mRNA expression of *CCL2*, a key chemokine responsible for activated HSC migration (Seki and Schwabe, 2015).

Hepatic stellate cells are well-known therapeutic targets for the treatment of liver fibrosis, and a series of antifibrotic drugs that target HSCs have been developed and tested in clinical trials (Higashi et al., 2017). A promising therapeutic strategy is to revert activated HSCs to a quiescent state. Although rottlerin was originally identified as an inhibitor of protein kinase C delta (PKCδ) (Gschwendt et al., 1994; He et al., 2019) and a recent report has shown that a general PKC inhibitor reduces a-SMA production in LPS-induced liver injury models in mice (Lee et al., 2013), a more detailed study has identified that rottlerin was an ineffective inhibitor of PKCδ activity, at least at micromolar concentrations (Soltoff, 2007). Systemically applied rottlerin was indicated to effectively treat experimental asthma via BK channel activation but without any effect on the control healthy mice (Goldklang et al., 2013). Here, we further demonstrated that systemically applied rottlerin has a marked antifibrotic effect in the liver through inhibition of HSC activation.

## Conclusion

In summary, we have demonstrated that activation of BK channels attenuates HSC activation by negatively regulating the profibrotic TGFβ1 signaling pathway. Our results not only link BK channels to profibrotic signaling pathways, but also provide evidence that pharmacological activation of BK channels may represent a promising therapeutic strategy for the treatment of liver fibrosis.

## Data Availability Statement

The datasets generated for this study can be found in the NCBI GEO database, accession number GSE139994 (https://www.ncbi.nlm.nih.gov/geo/query/acc.cgi?acc=GSE139994). The raw data supporting the conclusions of this article will be made available by the authors, without undue reservation, to any qualified researcher.

## Ethics Statement

The animal study was reviewed and approved by the Shanghai Jiao Tong University Institutional Animal Care and Use Committee.

## Author Contributions

LY, MZ, and BH performed the experiments and analyzed the data. Y-HW and MZ critically revised the manuscript. LY, Z-GZ, KH, and SH designed the research, conceived the ideas, and wrote the manuscript. All authors edited and reviewed the manuscript.

## Conflict of Interest

The authors declare that the research was conducted in the absence of any commercial or financial relationships that could be construed as a potential conflict of interest.

## Footnotes

1 http://www.geneontology.org/

2 http://www.bioconductor.org/

3 http://www.genome.jp/kegg/

4 http://www.biocarta.com/genes/index.asp

## Abbreviations

ACTA2, actin alpha 2: smooth muscle
BSA: bovine serum albumin
CCL2: C-C motif chemokine ligand 2
COL1A1: collagen type I alpha 1 chain
ECM: extracellular matrix
TGF β 1: transforming growth factor beta 1.
